# Molecular and Seroprevalence of *Mycoplasma gallisepticum* in Turkeys in Sylhet District of Bangladesh

**DOI:** 10.1002/vms3.70227

**Published:** 2025-02-25

**Authors:** Jahid Hasan Tipu, Rijon Miah, Obaidul Islam, Md. Mukidur Rahman, Lucky Talukdar, Rubel Miah, Md. Safwan Hussain, Md. Ashraful Islam, Md. Irtija Ahsan, Ahsan Raquib, Monira Noor

**Affiliations:** ^1^ Centre for International Health, Department of Global Public Health and Primary Care, Faculty of Medicine University of Bergen Bergen Norway; ^2^ Department of Pathology, Faculty of Veterinary, Animal and Biomedical Sciences Sylhet Agricultural University Sylhet Bangladesh; ^3^ Laboratory of Veterinary Epidemiology, College of Veterinary Medicine Chungbuk National University Chungbuk South Korea; ^4^ Department of Veterinary Sciences Chonnam National University Gwangju South Korea; ^5^ School of Public Health University of Saskatchewan Saskatoon Saskatchewan Canada; ^6^ Laboratory of Veterinary Laboratory Medicine, College of Veterinary Medicine Chungbuk National University Chungbuk South Korea; ^7^ School of Life Sciences University of Warwick Coventry UK; ^8^ Department of Epidemiology and Public Health, Faculty of Veterinary, Animal and Biomedical Sciences Sylhet Agricultural University Sylhet Bangladesh; ^9^ Department of Health Management, Atlantic Veterinary College University of Prince Edward Island Charlottetown Canada

**Keywords:** 16S rRNA gene, Diagnostic tests, *Mycoplasma gallisepticum* (MG), poultry, sensitivity, specificity, Sylhet

## Abstract

*Mycoplasma gallisepticum* (MG) poses a significant threat to Bangladesh's poultry industry, causing substantial economic losses every year. This study aimed to determine the prevalence of MG infection in turkeys using serum plate agglutination (SPA), enzyme‐linked immunosorbent assay (ELISA) and polymerase chain reaction (PCR) in Sylhet, Bangladesh from December 2019 to November 2020. In addition, we evaluated the diagnostic accuracy of these tests and identified potential risk factors associated with MG infection. A total of 250 blood samples and 250 tracheal swabs were collected from suspected turkeys across 25 farms from three sub‐districts of Sylhet namely Sylhet Sadar, Golapganj and Beanibazar. Blood samples were tested with SPA and ELISA, while tracheal swabs were analysed by PCR targeting the 16S rRNA gene of MG. The overall prevalence of MG was 35.2%, 29.2% and 25.6% for SPA, ELISA and PCR respectively. Higher infection rates were observed in turkeys aged 0–4 months (SPA 57.1%, ELISA 52%, PCR 42.8%), during winter (SPA 43.1%, ELISA 37.8%, PCR 30%) and among female turkeys (SPA 54.5%, ELISA 49.5%, PCR 45.5%). Geographically, the Beanibazar had the highest prevalence (SPA 54.2%, ELISA 48.6%, PCR 41.4%), compared to the Sylhet Sadar and Golapganj sub‐districts. Both SPA and ELISA tests showed 100% sensitivity, with specificity of 87.1% and 95.2%, respectively using PCR as a gold standard. Overall, these findings provide valuable insights for developing effective control measures for MG infections in the poultry industry of Bangladesh.

## Graphical abstract

We investigated *Mycoplasma gallisepticum* infection in turkeys using SPA, ELISA and PCR techniques in Sylhet, Bangladesh. Younger turkeys, females and winter season had higher prevalence rates, highlighting the key risk factors. SPA and ELISA demonstrated higher sensitivity but varied in specificity, offering crucial insights for effective detection and control strategies.

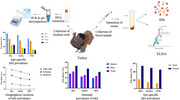

## 1 Introduction

Mycoplasmosis is one of the World Organisation for Animal Health (WOAH) notifiable avian diseases and is an emerging threat to the rising poultry industry worldwide due to noteworthy economic losses (Yadav et al. 2022). In turkeys, four species of *Mycoplasma* pathogens are well recognised: *Mycoplasma gallisepticum* (MG), *Mycoplasma meleagridis* (MM), *Mycoplasma iowae* (MI) and *Mycoplasma synoviae* (MS) (Abdelrahman et al. 2021; Kursa et al. 2024; Moalic et al. 1997; Wood and Wilson 2013). Among these, MG is recognised as one of the most economically significant pathogens due to its detrimental effects including increased feed conversion ratio, slower growth rates, reduced egg production, reduced hatchability, higher embryonic mortality and downgrading of carcasses (Dawood et al. 2022; Yadav et al. 2022). The presence of co‐infection with Newcastle disease (ND), infectious bronchitis (IB), influenza A, infectious laryngotracheitis (ILT), *Haemophilus paragallinarum* and *Escherichia coli* makes MG infection more severe (Samy and Naguib 2018; Zhang et al. 2024).

Clinical symptoms of MG infections include nasal discharge, tracheal rales, coughing, sneezing, infra‐orbital sinusitis and mild conjunctivitis (Mugunthan et al. 2023). Mycoplasmosis can enter a flock either through horizontal transmission, where the infection spreads via direct contact between birds, or through vertical transmission via contaminated eggs (Abdelrahman et al. 2021; Mugunthan et al. 2023; Yadav et al. 2022). However, the indirect transmission via humans or fomites may play a vital role due to the plausible persistence of MG in different environmental conditions, particularly cool, moist environments that extend survival on contaminated surfaces or equipment (Nagatomo et al. 2001). This emphasises the importance of biosecurity measures to minimise environmental contamination.

To eradicate the pathogen from a flock, rapid and reliable diagnostic tools are required to ensure that flocks are free of pathogenic *Mycoplasma*. The diagnosis of *Mycoplasma* has typically been carried out by serological tests: serum plate agglutination (SPA), enzyme‐linked immunosorbent assay (ELISA) and molecular‐based assays: polymerase chain reaction (PCR) (Emam et al. 2024; Kursa et al. 2024; Purba et al. 2023). Serological techniques are useful for monitoring flocks in MG control programs, but in some flocks, non‐specific reactions occur which may give false positive results (Ferguson‐Noel and Noormohammadi 2013). Molecular methods including detection, genotyping and strain differentiation are essential tools for accurately identifying *Mycoplasma* infections, which leads to a better understanding of the pathogen and its control (Bekő et al. 2019; Kursa et al. 2024).

In Bangladesh, turkey production is still at a primitive stage, with limited scientific studies addressing diseases in turkeys, including MG. Although no studies have quantified the specific impact of MG on turkey production, existing literature highlights a significant decline in turkey farming in the country, attributed to different challenges such as disease management (M. Hossain et al. 2024; Rashid et al. 2020; Shishir 2020). Therefore, this study aimed to fill this gap by exploring the prevalence of MG infection in turkeys in the Sylhet District of Bangladesh using SPA, ELISA and PCR techniques. Secondarily, we evaluated the sensitivity and specificity of these techniques to determine the diagnostic accuracy and identify the potential risk factors associated with the disease.

## 2 Materials and Methods

### 2.1 Study Area and Study Design

**FIGURE 1:**
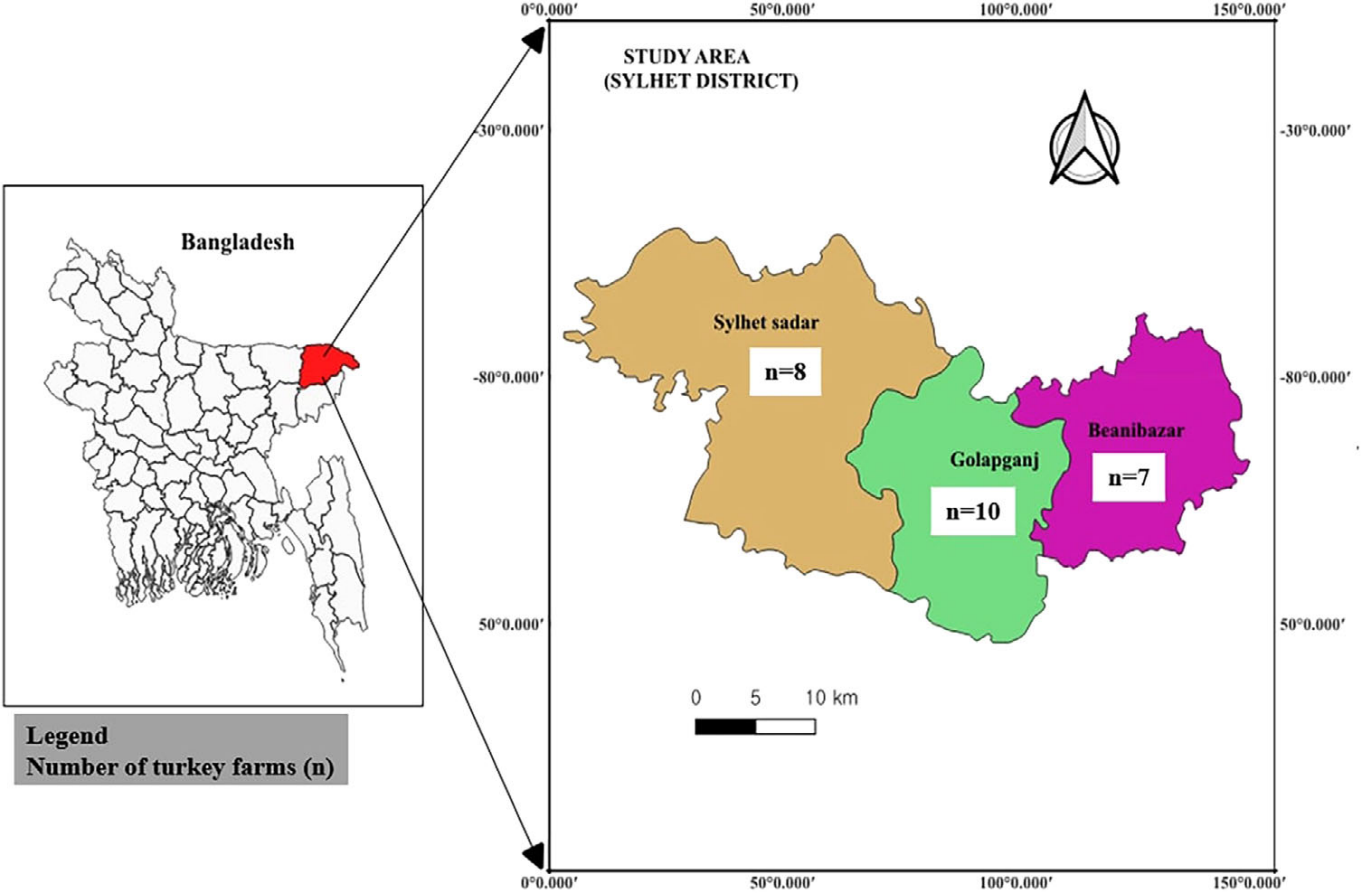
Map indicating the sampling sites (Beanibazar, Golapganj and Sylhet Sadar under the Sylhet District of Bangladesh).

This cross‐sectional study was conducted for 1 year from December 2019 to November 2020 in Sylhet Sadar, Golapganj and Beanibazar sub‐districts of Sylhet, Bangladesh (Figure 1). These locations were chosen due to the presence of turkey populations and their accessibility for sample collection.

### 2.2 Collection and Preparation of Samples

A total of 250 turkeys of different ages were selected randomly from 25 farms (Table S1). Initially, we contacted the Upazila Livestock Officer (ULO) to identify turkey farms within the sub‐districts. Farms were then selected randomly to ensure broad representation across the study area. From each selected farm, 10 turkeys were sampled and prioritised based on the clinical history of any respiratory signs, including dyspnoea, pronounced respiratory rales, sneezing, coughing, nasal discharge or reduced egg production in the flock. However, we also included apparently healthy birds in our sampling to ensure a comprehensive assessment of MG prevalence within the farms.

From the 250 selected turkeys, we collected a total of 500 samples comprising of 250 blood samples and 250 tracheal swabs, with each turkey providing both types of samples. For serological tests, blood samples were collected aseptically from the wing vein without anticoagulant, and the serum was separated in a sterile Eppendorf tube for subsequent analysis using both SPA and ELISA tests. For the PCR test, tracheal swabs were collected in a sterilised swab collection tube, mixed with phosphate‐buffered saline (PBS), and stored at −20°C to prevent degradation until the extraction of DNA.

### 2.3 Detection of MG Infection in Turkeys by Serological Test

#### 2.3.1 SPA Test

The SPA test was performed according to the instructions of the OIE Manual (Stear 2005). A volume of 0.02 mL of MG antigen (Lilli test MG RSA Antigen, Lillidale Diagnostics) and 0.02 mL of serum samples were mixed at room temperature for 2 min before reading the results. Positive reactions were observed as clumps within 2 min by three examiners and graded as +, ++ and +++ based on the clumped size to ensure consistency and reliability.

#### 2.3.2 ELISA

We conducted an indirect ELISA in a 96‐well plate utilising ID Screen *Mycoplasma gallisepticum* Indirect (Innovative Diagnostics, France) following the manufacturer's instructions. Samples were diluted at 1:50 with dilution buffer (5 µL of each sample with 245 µL of dilution buffer) in a pre‐dilution plate and the conjugate solutions (anti‐chicken‐HRP conjugate, 10X) were diluted at 1:10. Briefly, indirect ELISA followed standard protocol: We added the positive and negative controls, then pre‐diluted samples were added, incubated, washed and treated with conjugate and substrate solutions before recording optical density (OD) at 450 nm using an ELISA plate reader. For each sample S/P ratio and antibody titer were calculated using the following formula: A serum sample with S/P ratios of ≤ 0.5 and antibody titer < 843 is considered negative, while S/P ratios of ≥ 0.5 and antibody titer ≥ 843 were considered positive for MG infection, based on the cut‐off values provided by the kit used (Innovative Diagnostics, France).

$$S/P\;ratio=\frac{{OD}_{\mathrm{sample}}-{OD}_{\mathrm{nc}}}{{OD}_{\mathrm{pc}}-{OD}_{\mathrm{nc}}}$$

where nc is negative serum control and pc is positive serum control.

$$\mathrm{Antibody}\;\mathrm{titer}=\log_{10}\left(\mathrm{titer}\right)=0.91\times \log_{10}\left(\frac{S}{P}\right)=3.200$$

where titer = $10^{\log_{10}\mathrm{titer}}$.

### 2.4 Molecular Detection of MG Infection in Turkeys

#### 2.4.1 Extraction of DNA and PCR

The DNA was extracted from the tracheal swab samples using FavorPrep Blood Genomic DNA extraction mini kit, following the manufacturer's instructions (Favorgen Biotech Corp, Taiwan). Subsequently, we conducted a conventional PCR targeting the 16S rRNA gene of MG, as it is highly conserved across bacterial species, utilising previously published primers MG‐14F (GAGCTAATCTGTAAAGTTGGTC) and MG‐13R (GCTTCCTTGCGGTTAGCAAC) (Lauerman 1998). The reaction was conducted with a total volume of 50 µL, consisting of 25 µL master mix, 2.5 µL forward primer, 2.5 µL reverse primer, 15 µL RNase free water and 5 µL of template DNA. After mixing and settling using a mini centrifuge, the PCR reaction was performed under specific cycling conditions (initial denaturation at 94°C for 5 min, denaturation at 94°C for 30 s, annealing at 55°C for 30 s, extension at 72°C for 60 s, final extension at 72°C for 5 min with 40 cycles) to facilitate the amplification of the target sequence. To confirm the successful amplification of the targeted DNA fragment (expected amplicon size 185 bp), the amplified PCR products were analysed by 2% agarose gel electrophoresis using ethidium bromide (10 mg/mL). Finally, we ran the gel at 120 V for approximately 1 h and visualised the DNA amplicon under a UV documentation system (EDVotek, USA).

### 2.5 Data Management and Analysis

Detailed data for each turkey, including sampling date, age, sex, season and geographical location were recorded using a structured questionnaire at the time of sample collection. Turkeys were categorised into four age groups: 0–4, 4–8, 8–12 and > 12 months. The year was divided into three seasons to study seasonal variations: summer (March–June), rainy (July–October) and winter (November–February). Data were analysed using R software (version 4.2.2), with a *p* < 0.05 considered statistically significant. The chi‐square test was applied for the analysis of seasonal variation, sex‐specific distribution and regional prevalence of MG infection in turkeys, as detected by SPA, ELISA and PCR tests. In some cases, the results of Fisher's exact test were considered when more than 20% of the cells had expected frequencies less than five. In addition, to assess the diagnostic performance of SPA and ELISA tests, we calculated the sensitivity and specificity considering PCR as a gold standard test (Elyazeed et al. 2020) using the following formula:

$$\mathrm{Sensitivity}=\frac{\mathrm{True}\;\mathrm{positive}}{\mathrm{True}\;\mathrm{positive}+\mathrm{false}\;\mathrm{negative}}\times 100$$

$$\mathrm{Specificity}=\frac{\mathrm{True}\;\mathrm{negative}}{\mathrm{True}\;\mathrm{negative}+\mathrm{false}\;\mathrm{positive}}\times 100$$

## 3 Results

**FIGURE 2:**
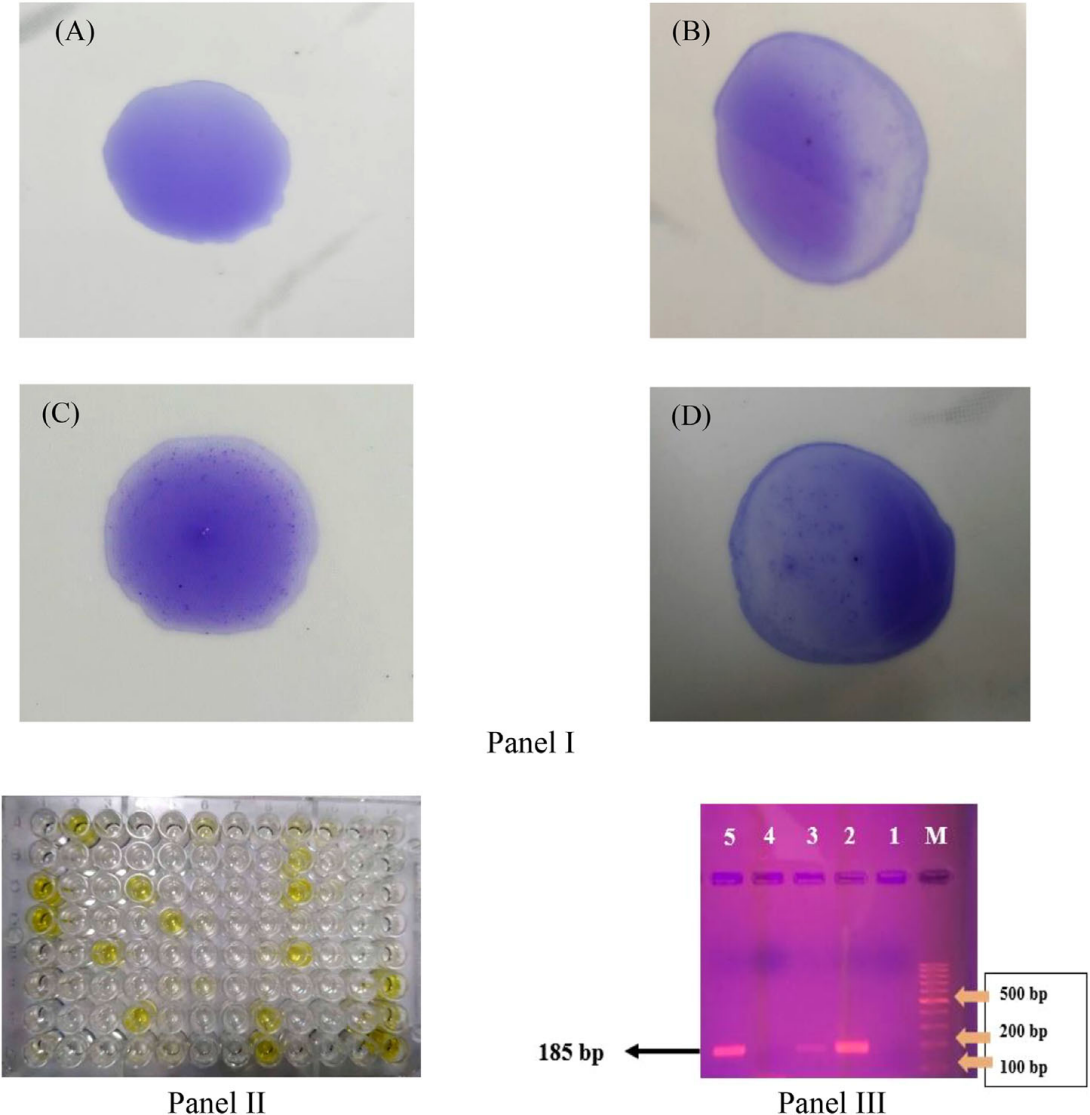
Panel I: Results of SPA test (A) no clumps formation (negative), (B) small clumps formation (+), (C) medium clumps formation (++) and (D) large clumps formation (+++). Panel II: Colour changed after adding stop solution in indirect ELISA (before recording the optical density (OD) at 450 nm in an ELISA plate reader machine. Panel III: Gel documentation of PCR product targeting the 16S rRNA gene of *Mycoplasma gallisepticum*. Lane M = 1 kbp DNA ladder, Lane 1 = negative control, Lane 2 = positive control and Lanes 3–5 = PCR products, with an expected band size of 185 bp.

Of the 250 blood and tracheal swab samples analysed, 88 tested positive (35.2%) in the SPA test, while 73 (29.2%) and 64 samples (25.6%) were positive in ELISA and PCR techniques respectively (Table 1). These results are also illustrated in Figure 2.

**TABLE 1:** Overall prevalence of MG infection in turkeys in the Sylhet District of Bangladesh during the study period.

Number of samples tested by each test	SPA		ELISA		PCR	
	Positive	Prevalence (%)	Positive	Prevalence (%)	Positive	Prevalence (%)
250	88	35.2	73	29.2	64	25.6

### 3.1 Age‐Specific Prevalence of MG Infection

**FIGURE 3:**
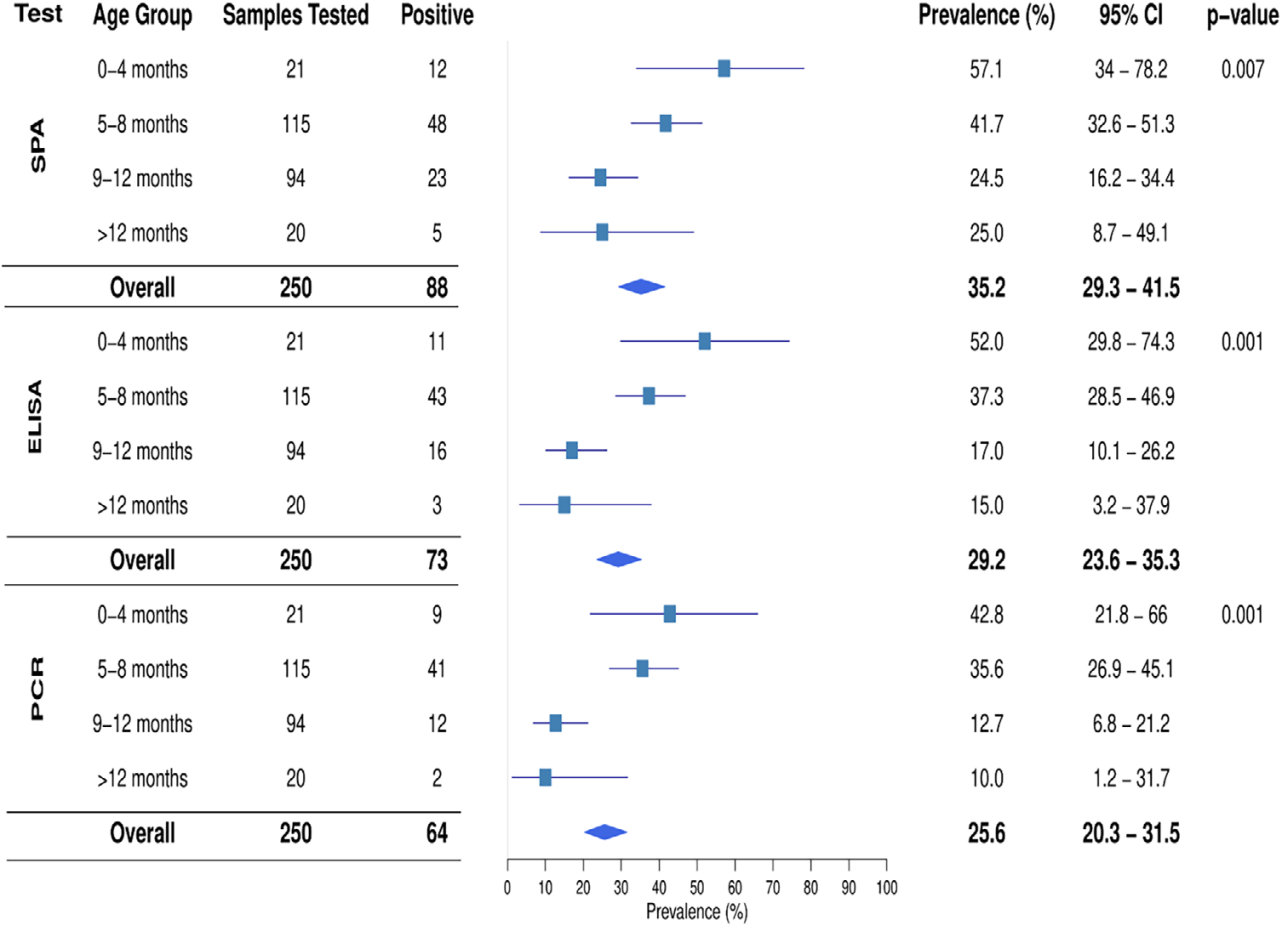
Prevalence of MG infection in turkeys across different age groups using SPA, ELISA and PCR tests during this study period in the Sylhet District of Bangladesh.

The prevalence of MG infection was accessed across different age groups of turkeys. The test result revealed a significant variation among the age groups (Figure 3), with the highest rate observed in turkeys aged 0–4 months (57.1%) and the lowest in those aged 9–12 months (25.0%). Similarly, the ELISA test indicated that turkeys 0–4 months of age had the highest prevalence, while the lowest was detected in birds older than 12 months. A similar trend was observed in the PCR results, confirming the age‐related prevalence pattern.

### 3.2 Prevalence of MG Infection in Turkeys Based on Sex

**FIGURE 4:**
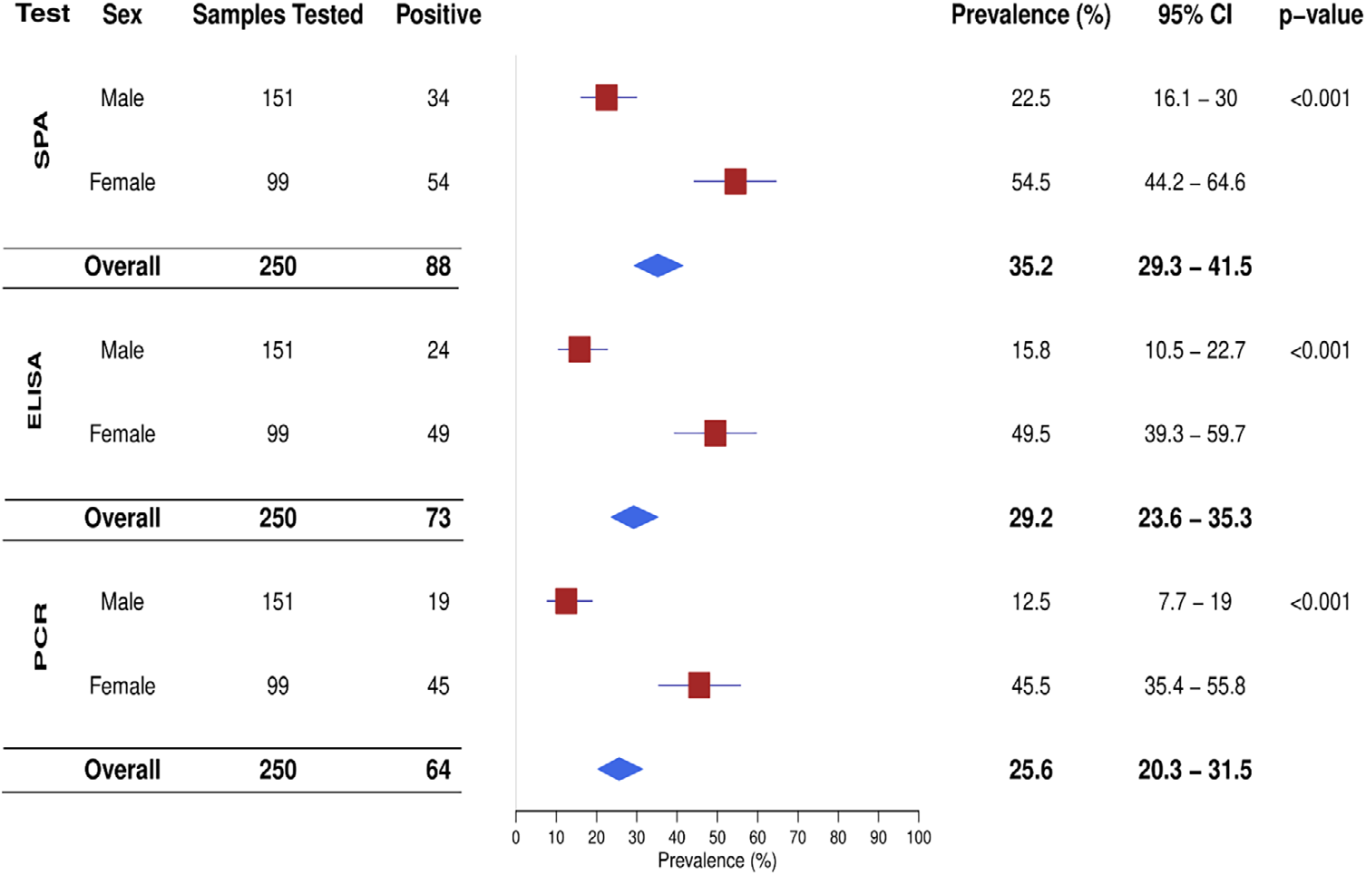
Sex‐specific distribution of MG infection in turkeys using SPA, ELISA and PCR tests during this study period in the Sylhet District of Bangladesh.

The study identified a significantly higher prevalence of MG infection in female turkeys across all diagnostic tests (Figure 4). According to the SPA test, MG prevalence was 54.5% in females compared to 22.5% in males. The ELISA test reported similar results, with a 49.5% prevalence in females and 15.8% in males. In addition, the results obtained by the PCR test were consistent with these findings, showing a prevalence of 45.5% in females compared to 12.5% in males.

### 3.3 Seasonal Variation of MG Infection in Turkeys

**FIGURE 5:**
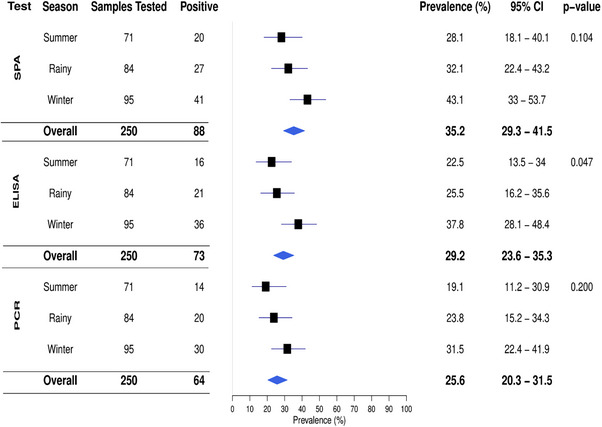
Seasonal variation in MG infection in turkeys as detected by SPA, ELISA and PCR tests during this study period in the Sylhet District of Bangladesh.

The prevalence of MG infection was highest in the winter season, followed by the rainy and summer across all tests (Figure 5). The SPA test showed a prevalence of 43.1% in winter, with the lowest at 28.1% in summer. Similarly, ELISA and PCR tests reported the highest prevalence in winter at 37.8% and 31.5% and the lowest in summer at 22.5% and 19.1%, respectively.

### 3.4 Geographical Distribution of MG Infection in Turkeys

**FIGURE 6:**
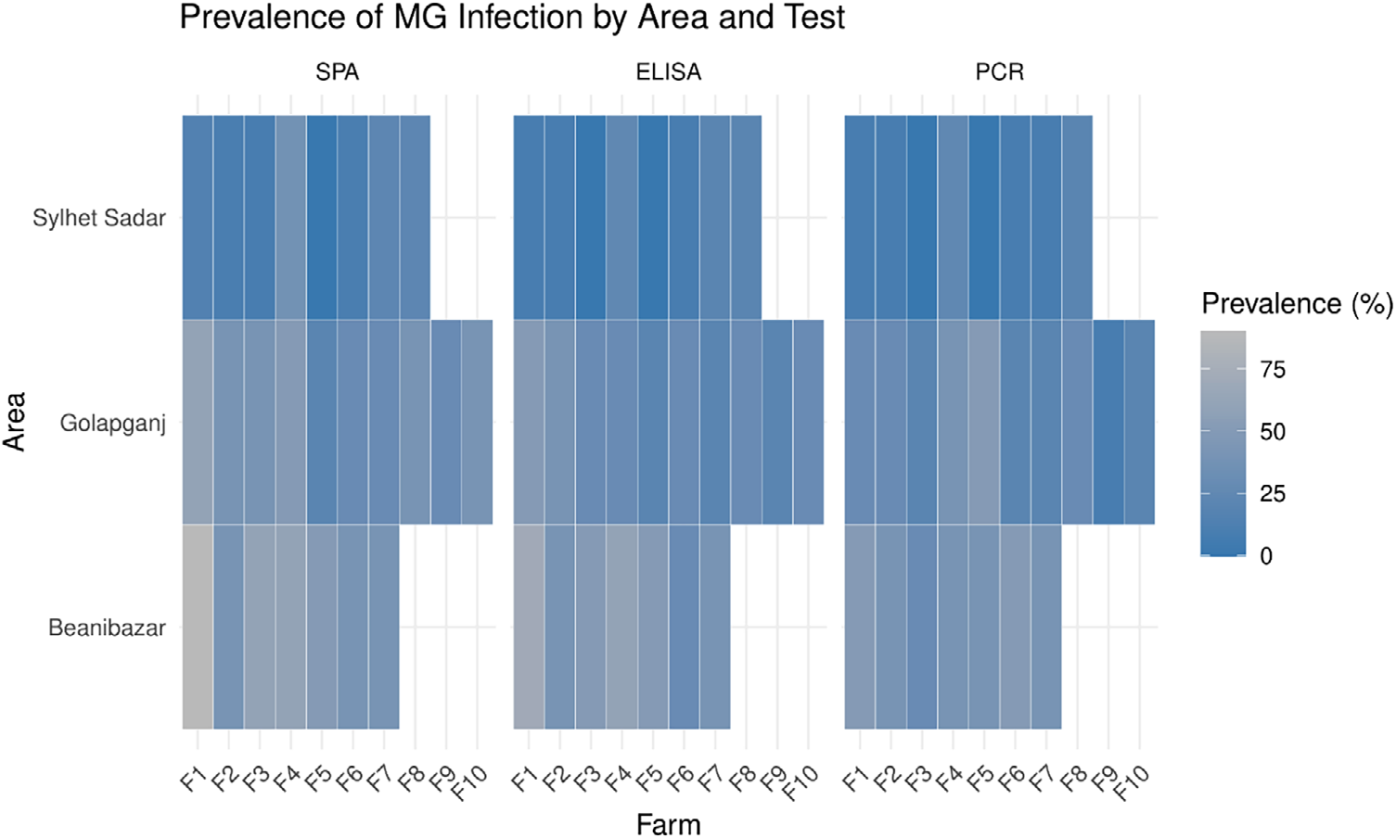
Prevalence of MG infection in the three sub‐districts of Sylhet by SPA, ELISA and PCR diagnostic tests during this study period.

The prevalence of MG infection varied significantly among the sub‐districts in Sylhet (Figure 6). Among these, Beanibazar emerged as the hotspot, and had the highest prevalence with 54.28% by SPA, 47.14% by ELISA and 34.28% by PCR test. In contrast, Sylhet Sadar reported a lower prevalence with only 10% by PCR, 11.25% by ELISA and 15% by SPA (Table S4).

### 3.5 Sensitivity and Specificity of SPA and ELISA Test

The sensitivity and specificity of the SPA and ELISA tests were evaluated considering PCR as the gold standard (Table 2). The sensitivity of both the SPA and ELISA tests was found to be 100%. However, the specificity differed between the two tests. The SPA test demonstrated a specificity of 87.1%, while the ELISA test had a higher specificity of 95.2%.

**TABLE 2:** Sensitivity and specificity of the diagnostic tests (SPA and ELISA) used in this study.

SPA test		ELISA test	
Sensitivity	Specificity	Sensitivity	Specificity
100%	87.1%	100%	95.2%

## 4 Discussion

The current study investigated the prevalence of MG infection in turkeys, examining associated risk factors such as age, sex, season and geographical location. Using three different diagnostic techniques (SPA, ELISA and PCR), we identified some important patterns that either align with or contrast findings from previous studies. The major differences are discussed in the following paragraph, including variations in diagnostic methods, risk factors and regional differences in the prevalence of MG infection.

The prevalence of MG infection in the present study ranged from 25% to 36% across three diagnostic techniques. MG prevalence has been reported to vary widely across countries and detection methods. For example, in Poland, rates of 8.33% were reported, with annual variations reaching up to 50% using PCR in floor‐reared birds (Kursa et al. 2024). In Iran, a prevalence of 33.3% was detected in broiler breeder farms using ELISA (Feizi et al. 2013), while in Algeria, 69.9% was observed using the SPA test in commercial poultry farms (Heleili et al. 2012). In South Africa, 15.55% was identified in five chicken breeds raised under an intensive system using PCR (Idowu, Mpofu, Zishiri, Adelabu, et al. 2024) and in India, 53.40% was reported in layers under an intensive system using ELISA (Udhayavel et al. 2016). These variations could be attributed to differences in diagnostic techniques, poultry breed/type and the geographic and genetic diversity of poultry populations.

We reported a higher prevalence of MG infection in younger turkeys (0–4 months), consistent with previous studies conducted in Bangladesh, Egypt and Algeria that reported a similar estimate in younger chickens (Heleili et al. 2012; K. M. Hossain et al. 2010; Islam et al. 2014b; Marouf et al. 2022). This could be attributed to the immature immune systems of the birds, which may render them more susceptible to infections (Benskin et al. 2009; Parkin and Cohen 2001). The key components of the innate immune response, such as macrophages and neutrophils are less developed in younger birds, potentially reducing their ability to combat infections effectively (Liu et al. 2024). Moreover, proteins like mannose‐binding lectin (MBL), which play a critical role in pathogen recognition and complement activation may also be under‐expressed in younger birds, further increasing susceptibility to MG infection (Idowu, Mpofu, Zishiri, Nephawe, et al. 2024). In contrast, some studies suggested that older chickens had a higher rate of MG infection, possibly due to prolonged exposure to MG, cumulative pathogen load or stress‐induced immunosuppression over time (Ayim et al. 2012; Islam et al. 2015). In addition, differences in species, sample size and sex‐related susceptibility could further contribute to these discrepancies. These findings highlight the complexity of age‐related dynamics in MG infection and suggest further research is needed to better understand these patterns, particularly in turkeys.

Our results also indicated a higher prevalence of MG infection in female turkeys compared to males. Comparable findings were reported in another study on chickens in model‐breeder poultry farms (Sarkar et al. 2005), which further consolidates our findings. We speculate that there may be a link to reproductive stress, particularly during the laying period when female birds experience a physiological strain, making them more vulnerable to infections (Dawood et al. 2022). Hormonal differences between males and females can also influence the immune response while differences in the physiology, behaviour and exposure to pathogens, potentially linked to management practices, may further explain this disparity (Klein 2000; Klein and Flanagan 2016; Pennell et al. 2012). Interestingly, we found that oestrogen—the female hormone—enhances immunity by binding to oestrogen receptors (ERα and ERβ) on immune cells, which boosts the proliferation and activity of macrophages, dendritic cells and T‐helper cells, thereby promoting a stronger immune response (Groothuis and Schwabl 2008; Harding and Heaton 2022). We recommended further studies to explore how reproductive stress and hormonal fluctuations impact susceptibility to MG infections, to better understand this disparity and inform poultry management practices.

Seasonal variation in MG prevalence was another key finding in the present study with a significantly higher prevalence during the winter season. This pattern aligns with previous studies conducted in different regions of Bangladesh, which also reported a higher MG prevalence in winter compared to other seasons (Ali et al. 2015; Islam et al. 2014a; Jalil and Islam 2010). Similarly, MG infection was more prevalent in the winter season as reported in studies from Egypt and Algeria (Heleili et al. 2012; Marouf et al. 2022). Several factors, such as environmental stress from the cold which may impair the immune systems of the turkeys, and the tendency for birds to be housed in closer quarters during colder months, could contribute to this seasonal increase (Oladokun and Sharif 2024; Saif 2009). Conversely, milder weather and better ventilation during summer likely reduces stress and the spread of pathogens, leading to lower prevalence rates (Abioja and Abiona 2021).

Geographically, the prevalence of MG infection was highest in the Beanibazar sub‐district compared to Sylhet Sadar and Golapganj. Variations in management and biosecurity practices might be the important reasons behind these findings (Chandiramani et al. 1966; Kursa et al. 2024). Other potential reasons could be the density of poultry flocks, local farming practices and environmental factors, which may account for these geographical differences (Nyoni et al. 2019). A previous study reported a higher prevalence of MG infection in poultry due to the higher density of farms in the Kishoreganj district of Bangladesh (Raquib et al. 2022). In contrast, the lower prevalence observed in Sylhet Sadar may be due to better management practices or the lower density of turkey populations (Famous et al. 2019). Further studies are required to investigate biosecurity practices and their relation to MG infection.

In this study, both SPA and ELISA tests demonstrated 100% sensitivity when compared to PCR, indicating that neither test missed any positive cases identified by the gold standard PCR test. Comparable findings were observed in a study conducted in Thailand (Wanasawaeng et al. 2015). While the high sensitivity is encouraging, it is important to consider that diagnostic tests rarely achieve 100% sensitivity in broader clinical or field conditions. The perfect sensitivity observed in this study may be attributed to the relatively small sample size, as smaller datasets may overestimate sensitivity due to sampling variability.

Despite the high sensitivity, the specificity of the SPA test was lower than that of the ELISA test, indicating that the SPA test may be more prone to false positives. This suggests that while both tests are suitable for detecting true positives, the ELISA test may be better suited for distinguishing true negatives (Han et al. 2024) and would be preferable considering the time and cost needed for a PCR test.

To the best of the author's knowledge, this is the first molecular study on MG infection particularly in turkey in Bangladesh that can provide an overview of the prevalence of MG infection in the turkey population of Bangladesh. The present study has several limitations to consider. There is a possibility of selection bias during the sample collection process. Our sampling approach, whether it be random or based on specific criteria, could inadvertently favour certain demographics or geographic regions over others In addition, the study was conducted during the COVID‐19 pandemic, a time when the agricultural sector in Bangladesh faced significant challenges (Islam et al. 2023), resulting in economic losses for farmers and leading to many farms closing or shifting to other types of poultry. Consequently, the number of available turkey farms for sample collection was limited, which constrained the sample size for this study and reduced the generalisability of the findings. Furthermore, the study focused only on a small number of variables, relied on a cross‐sectional design that limits causal inferences, and ignored numerous environmental and management factors. Moreover, sequencing of PCR‐positive isolates was not performed in this study, but it is recommended for future research as it might provide valuable insights into the genetic diversity and transmission dynamics of MG strains circulating in the population.

## 5 Conclusions

The present investigation highlights the prevalence of MG infection in turkeys in the Sylhet District of Bangladesh, with age, sex, season and geographical locations emerging as potential risk factors. The results obtained from SPA, ELISA and the gold‐standard PCR diagnostic tests suggest that younger birds, females and turkeys in colder season and certain sub‐districts are more susceptible to MG infection. Mycoplasmosis is a widespread issue potentially stemming from a lack of awareness among farmers regarding biosecurity and management practices. While providing valuable epidemiological insights, the study's limitations, such as the lack of sequencing of PCR‐positive isolates, underscore the need for further research to understand the genetic diversity of circulating strains and refine control measures for MG infections in turkeys.

## Author Contributions

**Jahid Hasan Tipu**: methodology, investigation, data curation, formal analysis, writing original draft, writing–review and editing. **Rijon Miah**: methodology, investigation, data curation, formal analysis, writing–review and editing, writing–original draft. **Obaidul Islam**: methodology, investigation, data curation, formal analysis, writing–original draft, writing–review and editing. **Md. Mukidur Rahman**: data curation, formal analysis. **Lucky Talukdar**: data curation, formal analysis. **Rubel Miah**: software, data curation, formal analysis, visualisation. **Md. Safwan Hussain**: software, data curation, visualisation, formal analysis. **Md. Ashraful Islam**: data curation, formal analysis. **Md. Irtija Ahsan**: software, data curation, formal analysis, visualisation, writing‐review and editing. **Ahsan Raquib**: software, data curation, visualisation, formal analysis. **Monira Noor**: conceptualisation, project administration, funding acquisition, supervision, resources, validation.

## Ethics Statement

The animal handling in this study was conducted following the Cruelty to Animals Act 1920 (Act No. I of 1920) of the Government of the People's Republic of Bangladesh. The study was approved by the Ethics Committee of Sylhet Agricultural University, Bangladesh.

### Peer Review

The peer review history for this article is available at https://publons.com/publon/10.1002/vms3.70227.

## Supporting information



Supporting information

## Data Availability

The data that support the findings of this study are available on request from the corresponding author. The data are not publicly available due to privacy or ethical restrictions.
