# Supplementary material for: Molecular and Seroprevalence of Mycoplasma gallisepticum in Turkeys in Sylhet District of Bangladesh
Source: Vet Med Sci. 2025 Feb 25;11(2):e70227. doi: 10.1002/vms3.70227 (PMC11855371; doi:10.1002/vms3.70227)
Supplement: Supplementary file 1 — Supporting information [file VMS3-11-e70227-s001.docx]

**Supplementary contents by Tipu et al., 2024**

**Table S1:** Sampling of MG infection at different subdistricts in Sylhet district of Bangladesh.

| **Location** | **Farm** | **Number of samples** | **Total** |
| --- | --- | --- | --- |
| Sylhet Sadar | F1 | 10 | 80 |
|  | F2 | 10 |  |
|  | F3 | 10 |  |
|  | F4 | 10 |  |
|  | F5 | 10 |  |
|  | F6 | 10 |  |
|  | F7 | 10 |  |
|  | F8 | 10 |  |
| Golapganj | F1 | 10 | 100 |
|  | F2 | 10 |  |
|  | F3 | 10 |  |
|  | F4 | 10 |  |
|  | F5 | 10 |  |
|  | F6 | 10 |  |
|  | F7 | 10 |  |
|  | F8 | 10 |  |
|  | F9 | 10 |  |
|  | F10 | 10 |  |
| Beanibazar | F1 | 10 | 70 |
|  | F2 | 10 |  |
|  | F3 | 10 |  |
|  | F4 | 10 |  |
|  | F5 | 10 |  |
|  | F6 | 10 |  |
|  | F7 | 10 |  |

**Table S2:** Sensitivity and specificity of the SPA test

| **Name of the test** | **PCR positive** | **PCR negative** | **Total** |
| --- | --- | --- | --- |
| **SPA positive** | 64 (true positive) | 24 (false positive) | 88 |
| **SPA negative** | 0 (false negative) | 162 (true negative) | 162 |
| **Total** | 64 | 186 | 250 |

Sensitivity of SPA test= TP/(TP+FN) = 64/ (64+0) = 100%

Specificity of SPA test = TN/(TN+FP) = 162/ (162+24) = 87.1%

**Table S3:** Sensitivity and specificity of ELISA test

| Name of the test | PCR positive | PCR negative | Total |
| --- | --- | --- | --- |
| ELISA positive | 64 (true positive) | 9 (False positive) | 73 |
| ELISA negative | 0 (false negative) | 177 (true negative) | 177 |
| Total | 64 | 186 | 250 |

Sensitivity of ELISA test= TP/(TP+FN) = 64/ (64+0) = 100%

Specificity of ELISA test = TN/(TN+FP) = 177/ (177+9) = 95.2%

**Table S4:** Prevalence of MG infection based on the geographical location

| **Area** | **Farm** | **No. of samples tested** | **Positive in SPA** | **Positive in ELISA** | **Positive in PCR** | **Prevalence**  **(%)** | | |
| --- | --- | --- | --- | --- | --- | --- | --- | --- |
|  |  |  |  |  |  | **SPA** | **ELISA** | **PCR** |
| Sylhet Sadar | F1 | 10 | 2 | 1 | 1 | 15% | 11.25% | 10% |
|  | F2 | 10 | 1 | 1 | 1 |  |  |  |
|  | F3 | 10 | 1 | 0 | 0 |  |  |  |
|  | F4 | 10 | 3 | 2 | 2 |  |  |  |
|  | F5 | 10 | 0 | 0 | 0 |  |  |  |
|  | F6 | 10 | 1 | 1 | 1 |  |  |  |
|  | F7 | 10 | 2 | 2 | 1 |  |  |  |
|  | F8 | 10 | 2 | 2 | 2 |  |  |  |
| Golapganj | F1 | 10 | 6 | 5 | 3 | 38% | 30% | 27% |
|  | F2 | 10 | 4 | 4 | 3 |  |  |  |
|  | F3 | 10 | 4 | 3 | 2 |  |  |  |
|  | F4 | 10 | 5 | 3 | 4 |  |  |  |
|  | F5 | 10 | 2 | 2 | 5 |  |  |  |
|  | F6 | 10 | 3 | 3 | 2 |  |  |  |
|  | F7 | 10 | 3 | 2 | 2 |  |  |  |
|  | F8 | 10 | 4 | 3 | 3 |  |  |  |
|  | F9 | 10 | 3 | 2 | 1 |  |  |  |
|  | F10 | 10 | 4 | 3 | 2 |  |  |  |
| Beanibazar | F1 | 10 | 9 | 7 | 5 | 54.28% | 48.57% | 41.42% |
|  | F2 | 10 | 4 | 4 | 4 |  |  |  |
|  | F3 | 10 | 6 | 5 | 3 |  |  |  |
|  | F4 | 10 | 6 | 6 | 4 |  |  |  |
|  | F5 | 10 | 5 | 5 | 4 |  |  |  |
|  | F6 | 10 | 4 | 3 | 5 |  |  |  |
|  | F7 | 10 | 4 | 4 | 4 |  |  |  |
